# Web-Based Conversations Regarding Fathers Before and During the COVID-19 Pandemic: Qualitative Content Analysis

**DOI:** 10.2196/40371

**Published:** 2023-02-15

**Authors:** Lindsay Bouchacourt, Mike Henson-García, Kristen Leah Sussman, Dorothy Mandell, Gary Wilcox, Michael Mackert

**Affiliations:** 1 Stan Richards School of Advertising and Public Relations The University of Texas at Austin Austin, TX United States; 2 School of Public Health The University of Texas Health Science Center - Dallas Regional Campus Dallas, TX United States; 3 School of Journalism and Mass Communication Texas State University San Marcos, TX United States; 4 School of Community and Rural Health University of Texas Health Science Center at Tyler Tyler, TX United States; 5 Center for Health Communication The University of Texas at Austin Austin, TX United States

**Keywords:** social media, expecting fathers, new fathers, Twitter, Reddit, content analysis, topic model, topic analysis, parent, father

## Abstract

**Background:**

Studies of new and expecting parents largely focus on the mother, leaving a gap in knowledge about fathers.

**Objective:**

This study aimed to understand web-based conversations regarding new and expecting fathers on social media and to explore whether the COVID-19 pandemic has changed the web-based conversation.

**Methods:**

A social media analysis was conducted. Brandwatch (Cision) captured social posts related to new and expecting fathers between February 1, 2019, and February 12, 2021. Overall, 2 periods were studied: 1 year before and 1 year during the pandemic. SAS Text Miner analyzed the data and produced 47% (9/19) of the topics in the first period and 53% (10/19) of the topics in the second period. The 19 topics were organized into 6 broad themes.

**Results:**

Overall, 26% (5/19) of the topics obtained during each period were the same, showing consistency in conversation. In total, 6 broad themes were created: fatherhood thoughts, fatherhood celebrations, advice seeking, fatherhood announcements, external parties targeting fathers, and miscellaneous.

**Conclusions:**

Fathers use social media to make announcements, celebrate fatherhood, seek advice, and interact with other fathers. Others used social media to advertise baby products and promote baby-related resources for fathers. Overall, the arrival of the COVID-19 pandemic appeared to have little impact on the excitement and resiliency of new fathers as they transition to parenthood. Altogether, these findings provide insight and guidance on the ways in which public health professionals can rapidly gather information about special populations—such as new and expecting fathers via the web—to monitor their beliefs, attitudes, emotional reactions, and unique lived experiences in context (ie, throughout a global pandemic).

## Introduction

When studying expecting parents, studies have predominantly focused on the mother, leaving a large gap in knowledge about expecting fathers. However, studies have shown that men want to be involved in their partner’s pregnancy, but they struggle with knowing how to be involved [1]. Others criticize that the prenatal health care system is not set up to support the father’s involvement [2]. Thus, more studies are needed to understand how to effectively reach out to and connect with new and expecting fathers. A medium to reach this audience is social media. Social media is an effective tool to communicate with an audience, and by studying web-based conversations, researchers can understand what individuals care about and want to share with the world. Social media is a useful tool to understand people’s experiences, and in a health context, the insights obtained from social media analyses can be used to guide public health messages and efforts [3-5].

Studies analyzing fathers and their use of social media and web-based platforms have explored how different types of fathers use different sites including Black fathers using Facebook [6], Swedish first-time fathers writing blogs [7], and fathers using Reddit for peer support [8]. This study took a broad approach by capturing publicly available social media posts (Twitter and Reddit) that are related to being or becoming a new or expecting father, thus capturing posts that originate not only from fathers but also from other close ties. By casting a broad net, we contribute to a more heterogeneous understanding of the web-based content surrounding new and expecting fathers and enrich the research on expecting fathers. This study is also unique in that it compares 2 periods to see if there has been a change in web-based conversation owing to the advent of the COVID-19 pandemic. The social media posts that were collected for the analysis were shared by either a new or expecting father or an account discussing content related to being a new or expecting father. The findings provide insight into the web-based behavior of new and expecting fathers, along with the accounts interacting with this group. Therefore, the following research question (RQ) was proposed:

*RQ1*: What topics emerge from the web-based conversations surrounding new and expecting fathers?

Social media data were collected over a 2-year period—between February 1, 2019, and February 12, 2021. In the middle of this period, the COVID-19 pandemic was declared as a global health emergency, affecting all aspects of life, including parenting. During the pandemic, parents faced unique challenges, including childcare and education [9]. Owing to the many changes, challenges, and heightened stress that parents faced and continue to face during the pandemic [10], we decided to split the data and compare the periods of 1 year before the pandemic and the first year of the pandemic. Thus, our second RQ was the following:

*RQ2*: Do the web-based conversations from before the pandemic differ from those during the pandemic?

The findings from the study can guide public health communication strategies regarding how to reach new and expecting fathers via the web. By understanding what fathers and other relevant accounts post on the web, communication professionals—either public health or marketing and advertising professionals—will be able to better connect and communicate with fathers.

## Methods

### Data Acquisition

The data set for this study was obtained using Brandwatch, a social media listening software that is designed to collect publicly shared posts, comments, and web-based conversations [11]. Twitter and Reddit data were included as sources for data collection using Brandwatch. Although Twitter and Facebook have gained more attention in the literature, Reddit attracts >330 million active users and serves as a source for sufficient data collection [12]. Next, using Brandwatch’s query editor, the authors wrote queries by combining simple terms to form a Boolean expression [13] based on social media mentions and language relating to experiences of new fatherhood (eg, “becoming a father”). The variations were selected based on common English adverbs and adjectives that could be associated with a person using first-person reference to becoming a father. Filters were applied to collect English-language posts that excluded profanities, pornographic content, promotions, and giveaways. Then, using a manual process of trial and error, the language in the returned posts was reviewed for accuracy and to mitigate noise in the data set. Irrelevant conversations were identified and then removed using negation operators (eg, NOT “I miss him greatly” NOT “Almighty” NOT “begotten” NOT “cops”) until the results appeared to be consistent in quality and relevance. The query operators and examples used in the study are shown in Table 1, and the entire Boolean code is provided in Multimedia Appendix 1.

The full data set returned 122,663 English-language conversations that occurred between February 1, 2019, and February 12, 2021. Then, the data set was cleaned using *R*, removing all duplicate posts (ie, retweets) and retaining variables of interest, before being divided into 2 separate data sets of conversations that occurred either *before* or *after* the pandemic was announced as a global health emergency by the World Health Organization on January 31, 2020 [14]. After removing duplicates, the corpus included 44,335 relevant posts. Next, after removing *organizational* posts (eg, posts from an organization), the final clean data set included 42,298 observations, with 18,303 (43.27%) occurring before and 23,995 (56.73%) occurring after the announced pandemic. The final data set included a sample of 37.12% (8907/23,995) Reddit observations and 62.88% (15,088/23,995) Twitter observations collected after the pandemic had been announced, a 31% increase in mention volume from the first data set collected before the pandemic, which contained 25.96% (4752/18,303) Reddit observations and 74.04% (13,551/18,303) Twitter observations. The 18,303 posts that occurred before the pandemic comprised 14,622 unique users, with an average of 1.246 (SD 2.161) posts per individual. The second data set resulted in a similar ratio, with 18,710 unique users making up 23,995 posts, with an average of 1.276 (SD 2.519) posts per individual*.*

As was provided by the web-based location metadata of the users in this sample, before the pandemic, of the 19,272 posts, approximately 11,988 (62.2%) posts originated in North America, 3439 (17.84%) originated in Europe, 1780 (9.24%) originated in Africa, 1422 (7.38%) originated in Asia, 514 (2.67%) originated in Australia and Oceania, and 129 (0.67%) originated in South America. Regarding the during-pandemic users, the sample containing 23,995 posts was summarized in the same ordinal fashion, with similar geographical distribution of approximately 13,582 (56.6%) posts originating in North America, 5003 (20.85%) originating in Europe, 2843 (11.85%) originating in Africa, 1965 (8.19%) originating in Asia, 336 (1.4%) originating in Australia and Oceania, and 265 (1.1%) originating in South America. The geolocation information has also been summarized and provided in Table 2.

**Table 1:** Query operators^a^.

Description	Operator	Example	Result
Basic	The *QUOTES* operator	“becoming a father**”**	Finds mentions of the exact phrase, “becoming a father”
Basic	The *OR* operator	#newdad *OR* #expectantfather	Finds mentions of #newdad or mentions of #expectantfather
Complex	The *NEAR/n* operator	(“I am” OR “I will”) *NEAR/10* (“be a father” OR “becoming a dad”)	Finds mentions of “I am” within 10 words of “becoming a dad” or “be a father” and mentions of “I will” within 10 words of “becoming a dad” or “be a father”
Complex	The wildcard operator	“expect* dad”	Finds mentions with the root word, *expect*; eg, expecting dad and expectant dad

^a^The full query is provided in Multimedia Appendix 1.

**Table 2:** Summary of user posts according to location.

Source location	Posts before the pandemic (n=18,303), n (%)	Posts during the pandemic (n=23,995), n (%)
North America	11,385 (62.2)	13,583 (56.61)
Europe	3266 (17.84)	5003 (20.85)
Africa	1691 (9.24)	2843 (11.85)
Asia	1350 (7.38)	1965 (8.19)
Australia and Oceania	488 (2.67)	336 (1.4)
South America	122 (0.67)	265 (1.1)

### Text Analysis

During the next step in the text analytic process, the 2 samples were analyzed using SAS Text Miner (version 15.1; SAS Institute) [15] through natural language processing and machine learning techniques to identify otherwise hidden themes in the conversations. SAS Text Miner provides the ability to parse and extract information from text, filter and store the information, and assemble tweets into related topics for introspection and to obtain insights from the unstructured data [16]. First, the text topic node was used to combine terms into topic groups. Then, a text filter node was used to exclude words that appeared in <4 messages, as a conservative measure to reduce noise. The parsing process handled by the software involves sorting all the words into separate terms and assigning a numerical identifier to them. Words that are not essential (“of,” “and,” and “but”) are removed. After the software completes this process, the filter feature allows the researcher to review the output and remove unrecognizable characters and strings of letters. A single reviewer followed a systematic process to maintain objectivity. The same process has been used in multiple studies examining RQs related to public reactions, health, and other issues across social media.

SAS Text Miner sorted the posts into topics, and the authors agreed on a 9-topic solution for period 1 (before the pandemic) and a 10-topic solution for period 2 (during the pandemic). To interpret and make sense of each topic, a random sample of approximately 30 posts per topic was manually assessed. An author performed the initial assessment, and then, the interpretations were discussed among all authors until consensus was reached. Next, the final 19 topics were manually sorted into 6 broad themes based on similar characteristics and patterns of the posts within each topic and the overall subject matter of the topic.

Finally, a sentiment analysis of the posts was performed. Analysis of emotion words in textual data was performed using the R package, *Syuzhet*, and the *get_nrc_sentiment* function. The packages implement the National Research Council Emotion Lexicon by Saif Mohammad, which comprises 8 basic words of emotion expressions for anger, fear, anticipation, trust, surprise, sadness, joy, and disgust [17].

### Ethical Considerations

Ethics approval was not needed for this study, as public social media posts are considered as part of the public domain. Analysis of these public posts is not considered to be human participants research.

## Results

### Overview

The data revealed that 47% (9/19) of the topics appeared between February 1, 2019, and January 30, 2020 (before the pandemic), and 53% (10/19) of the topics appeared between January 31, 2020, and February 12, 2021 (during the pandemic). The number of mentions, the topics, a brief description, and an example social media mention are shown in Table 3 for the *prepandemic* period and in Table 4 for the *during-pandemic* period. Additional topic analysis results are also presented visually using the R package, *wordcloud2*, in Multimedia Appendices 2 and 3.

The first 5 topics across both periods were the same (father readiness, interpersonal troubles, Father’s Day, best dad proclamations, and pregnancy announcements). The remaining topics were unique across the 2 periods. The 19 topics were divided into 6 broad themes: fatherhood thoughts, fatherhood celebrations, advice seeking, fatherhood announcements, external parties targeting fathers, and miscellaneous. The themes and their corresponding topics are described in the following sections.

Differences between the 2 groups were further examined using sentiment analysis. The analysis measured anger, fear, anticipation, trust, surprise, sadness, joy, and disgust. The results are shown in Multimedia Appendix 4.

**Table 3:** Prepandemic topics, descriptions, and examples.

ID	Topic	Description	Example	Number of posts (n=15,408), n (%)
Pre1	Fatherhood readiness	Users share whether they are ready to be a father	“People ask me if I’m ready to be a father, and I say I’m as ready as I will be.”	2475 (16.06)
Pre2	Interpersonal troubles	Users share interpersonal troubles between them and their expectant partner or parent of their child, hoping for advice	“I have been dating [removed for privacy] for around 2 years. Marriage and kids was always discussed, but I never felt like it was the right time for either...6 months ago she announced she was pregnant.”^a^	2329 (15.12)
Pre3	Father’s day	Users celebrate Father’s Day on the web	“I am proud to be a father today, and every day. Happy #FathersDay!”	2232 (14.49)
Pre4	Best dad proclamations	Users claim that they will be the best father	“And I promise for my child I will be the best father a kid could ask for.”	1235 (8.02)
Pre5	Pregnancy announcements	Users share their pregnancy news on the web	“I’m gonna be a dad!!!”	1654 (10.73)
Pre6	New father celebration	Users celebrate becoming a father	“I’m gonna be a father. This is not a drill. I’m gonna be a father.”	1261 (8.18)
Pre7	Expecting parent content	Content originating from or targeting expecting parents	“Pregnancy Definitions: Here are 122 of the top pregnancy terms pregnant parents need to know!”	1543 (10.01)
Pre8	Becoming a father again	Fathers announce that they are going to be a father again	“I’m going to be a father in a few months. Am I ready? YES!”	664 (4.31)
Pre9	Newborn-related articles	Links to external articles about various topics about newborns	“About newborn sleep [link removed for privacy].”	2015 (13.08)

^a^For brevity, this example includes only the first 3 sentences of the post.

**Table 4:** During-pandemic topics, descriptions, and examples.

ID	Topic	Description	Example	Number of posts (n=18,745), n (%)
Dur1	Fatherhood readiness	Users state whether they are ready to be a father	“I am ready to be a husband but not ready to be a father.”	2253 (12.02)
Dur2	Interpersonal troubles	Users share interpersonal troubles between them and their expectant partner or parent of their child or issues regarding pregnancy, hoping for advice	“Firstly, I know professional help is needed, I just want to get some feedback here, please. 38 y/o male. Wife {34 y/o} and I have been married for about 7 years...We never really discussed having kids vs. not having kids until after we were married, but the understanding was kind of always that we would...or at least I thought that’s what it was.”^a^	2903 (15.49)
Dur3	Father’s day	Users celebrate a happy Father’s Day	“I am blessed to be a father. Happy Father’s Day to all the father figures out there.”	2846 (15.18)
Dur4	Best dad proclamations	Users state that they will be the best father	“I will be the best dad I can be.”	1612 (8.59)
Dur5	Pregnancy announcements	Users share that they are going to be a father	“Hey everybody! I’m gonna be a dad!”	1934 (10.32)
Dur6	Pregnancy congratulations	Users congratulate other users for their pregnancy announcements	“Awwwwwww congratulations.”	953 (5.08)
Dur7	New dad life	Posts from new fathers experiencing life with their new baby	“Is it the weekend yet? Oh wait, I have kids!”	2794 (14.91)
Dur8	Account replies	An account called Family Core is replying to users’ posts	“If YOU are EXPECTING...We are here to HELP Join The Family Core today.”	436 (2.33)
Dur9	Comedic replies	Comedic replies to other users’ posts	“I am available to be your new dad, if you would like.”	2493 (13.29)
Dur10	Financial help requests	Replies to other posts, asking for financial help for a small-scale business loan	“Good day! Please I need your help financially, any amount so I can start up a small scale business for myself and be able to help my family. I’m going to be a father in few months and I don’t have a job. Please I really need help financially please.”	521 (2.78)

^a^For brevity, this example includes only the first 3 sentences of the post.

### Fatherhood Thoughts

This theme consisted of the largest number of topics. Fathers used social media as a place to share their thoughts on fatherhood and fatherhood-related topics. Pre1 and Dur1 focused on fatherhood readiness. Pre1 consisted of social media mentions (often replies to other people’s posts) that state “I am ready to be a father” or “I am not ready to be a father.” Dur1 included users sharing their readiness (or lack thereof) for being a father. The posts were about users who are exclaiming that they are ready to be a father. Pre4 and Dur4 focused on fathers proclaiming that they will be the “best dad.” Pre4 included users exclaiming that they will be the best father they can be for their child. Some users did not have children yet, but said that when they do have kids, they will strive to be the best father they can be. In Dur4, the posts showed users stating that they will be “the best dad” or “the best dad in the world.” Some posts tagged another user, so that the tagged user will see the post, thus making it more conversational and interactive. Dur7 included posts from new fathers experiencing life with their new baby and sharing these experiences on the web.

### Fatherhood Celebrations

Many fathers and other users used social media to celebrate different aspects of fatherhood, including celebrating Father’s Day and becoming a father. Pre3 and Dur3 focused on celebrating Father’s Day on the web. Both topics captured straightforward posts of users celebrating Father’s Day. Families were thanking the fathers and father figures in their lives, and fathers were thanking their families for wishing them a happy Father’s Day. Fathers also posted about being lucky to be a father to their children. Pre6 included users celebrating becoming a father. Users shared their excitement on finding out that they are going to be a father. Some users also shared their excitement about public figures and celebrities becoming fathers. Dur6 showed users congratulating other users for their pregnancy announcements, using the word “congratulations.”

### Advice Seeking

Some fathers wanted to use the web space to seek advice about pregnancy and parent-related situations. Pre2 and Dur2 focused on interpersonal troubles of users who are expecting or have children. These topics were unique in that the social media mentions originate from Reddit, which allows for long posts. Pre2 and Dur2 included social media mentions from Reddit of users sharing personal stories about a situation in their life regarding being pregnant, having a pregnant partner, or having children. Users made these posts seeking advice for their situation. Advice seekers wrote about a conflict that had been occurring between the 2 expecting parents. Some of the posts from Pre2 included posts about a man whose fiancée had a miscarriage, a woman with 4 children whose partner left after her last pregnancy, and a man whose partner is pregnant but the 2 of them have been struggling to stay together. Some of the posts from Dur2 included posts about a woman who is pregnant and will be a single mother and a man who is in a long-term relationship and does not want children, whereas his partner does.

### Fatherhood Announcements

Overall, 16% (3/19) of the topics consisted of fathers using social media to share father-related news. Pre5 and Dur5 captured pregnancy announcements on the web. Both topics included straightforward posts of users exclaiming “I’m gonna be a dad!” Pre8 captured posts with fathers announcing that they are going to be a father again. The posts were either from users announcing that they will be a father again or focused on announcements of a celebrity being a father again.

### External Parties Targeting Fathers

In this theme, the topics consisted of social media mentions that included content related to pregnancy and newborns. Pre7 captured content originating from or targeting expecting parents. Some social media posts included content that originated from companies that are selling newborn-related products, such as milestone blankets and pregnancy books. Some posts included *parent cheat sheets*, which included links to external articles that provide baby-related and parenting advice. Other posts showed users announcing that they are expecting a baby and that they are a “mom to be” or a “dad to be.” Pre9 included links to external articles about various topics regarding newborns. The articles included information about newborn sleep, preventing colic, baby’s stress, reading with newborns, and newborn brain development. Dur8 included replies from an account called Family Core. Family Core was replying to other users’ posts and promoting their account by stating that “they can help if you are expecting.”

### Miscellaneous

Overall, 11% (2/19) of the topics (Dur9 and Dur10) that were captured included social media mentions that do not directly fit with the other fatherhood-related topics. This theme only consisted of topics that were present during the pandemic. Dur9 captured comedic replies to other users’ posts. The replies included the word “dad” (eg, “I am your new dad.”). This was typically a comedic reply in response to a post in which another user would have benefited from having the replier as “their dad.” Dur10 captured replies from an account that is replying to other users’ posts (often the posts of Senators or Congress people), asking for financial help through a small business loan. This user was asking for financial help because they are going to be a father in a few months.

## Discussion

### Principal Findings

This study examined web-based conversation originating from or related to new and expecting fathers. The study examined social media mentions over a 2-year period—1 year before the COVID-19 pandemic and 1 year during the pandemic. During the 2 periods, much of the conversation was similar, as approximately half of the topics (10/19, 53%) overlapped with each other. Overall, the findings show that new and expecting fathers used social media to share good news, celebrate fatherhood, seek advice, and interact with other fathers. Other relevant accounts used social media to advertise baby products to fathers and promote baby-related articles to fathers.

The similarity in half of the topics (10/19, 53%) showed the consistency in the web-based conversations of new and expecting fathers. This 53% (10/19) of the topics (father readiness, interpersonal troubles, Father’s Day, best dad proclamations, and pregnancy announcements) related to content originating from a new or expecting father, as opposed to content being targeted to fathers from companies or websites. This 53% (10/19) of overlapping topics showed what fathers are sharing on the web both before and during the pandemic and that they tend to maintain similar interests.

The topic covering *interpersonal troubles* provided the deepest insight into struggles that expecting fathers face, especially regarding their pregnant partner. These posts were obtained from Reddit. Compared with Twitter, Reddit is perceived by users as a more private space on the web, and the subreddit where the posts came from focused on advice seeking. Users shared their personal stories, with the hope that other users may reply and provide advice and guidance. Studies show that sharing information on the web can be therapeutic and cathartic, especially during a crisis [18]. Ammari and Schoenebeck [19] interviewed fathers to understand their relationship with social media and found that men have concerns regarding privacy and judgment when it comes to posting about their children on the web. Reddit may act as a safe space for fathers to remain anonymous and share their struggles without fear of judgment. The difference in use between Twitter and Reddit can be explained through the theory of uses and gratifications [20], which is used to understand why people use certain types of media by examining the needs people have when using a media type and the gratifications people get from using the media. New and expecting fathers seem to use Twitter to share announcements and celebrate fatherhood, whereas Reddit can be used when fathers are seeking advice for a problem—particularly when they may prefer anonymity. Future studies should consider other media channels that fathers rely on and what needs those channels satisfy.

Strong emotions related to the transition to fatherhood—such as trust, joy, and anticipation—were often expressed by new and expecting fathers in both prepandemic and during-pandemic phases of the analysis. Interestingly, compared with posts in the period before the pandemic, posts during the pandemic had decreased levels of joy and increased levels of fear and sadness, which can represent the difficult nature of this period. Fatherhood comes with challenges, and the pandemic produced unique stressors for parents, which would certainly result in experiencing negative emotions. Emotional analysis provides insight into potential attitudes and cognitions of fathers, as emotions affect attitudes and behavior. Moreover, in both study periods, fathers used the web to share advice, seek social support, and connect with others dealing with similar situations and interpersonal troubles. The need for social support is important for new and expecting fathers [2]. It would be worthwhile for future studies to examine the utility of leveraging these emotional appeals in health communication efforts regarding childbirth, child rearing, and parenthood, along with incorporating the importance of social support. In addition, future studies should examine whether there are substantial differences in emotions expressed on the web according to the platform (ie, Twitter vs Reddit vs Facebook).

Overall, the findings outlined in this paper tend to diverge from previous scholarship examining fatherhood-related conversations that occur within social media environments. For instance, by using a latent topic modeling approach to data stemming from the Daddit subreddit, Ammari et al [19] uncovered 3 topics from the data including (1) conversations related to experiences in the neonatal intensive care unit, (2) Halloween and Halloween costumes, and (3) legal challenges experienced by users. However, none of these topics explicitly materialized in our analysis. Sepahpour-Fard et al [21] also applied topic modeling techniques to characterize the parenting conversations on parenting subreddits—including the Daddit subreddit—and out of the 12 latent topics that were reported in the paper, only 1 topic re-emerged in this study. Specifically, there is an interesting similarity in sentiment between the *thank you and appreciation* theme determined by Sepahpour-Fard et al [21] and the *pregnancy congratulations* theme that we found in our analysis. The overlap in these findings point to and underscore the positive emotions that are experienced in the transition to fatherhood, despite considerable societal change. This finding is further bolstered by the results from our sentiment analysis, which determined that trust and joy were the top 2 emotions expressed in web-based conversations surrounding fatherhood, throughout the transition from a prepandemic society to a postpandemic society. It is important to note that unlike our analysis, which used data from both Reddit and Twitter platforms, the studies by Ammari et al [19] and Sepahpour-Fard et al [21] solely analyzed Reddit data, which may partially explain why the study findings diverged so drastically. Furthermore, relative to previous studies, themes of interpersonal troubles are highly represented in our analysis, which may serve as a digital signature indicative of increased pandemic-related stressors experienced by new fathers and their growing families.

Other scholars have used social media analyses to examine changes in emotion and sentiment as societies across the globe grappled with and responded to the COVID-19 pandemic. For instance, using data retrieved from Twitter, several textual and sentiment analyses have been conducted to explore health care providers’ emotional reactions during the global disaster, with studies finding that, in general, levels of positive emotions (eg, joy) have decreased over time, whereas levels of negative emotions (eg, sadness and disgust) have increased [22,23]. However, specific to the emotional experiences of expecting and new fathers, a few qualitative studies have reported that fathers experienced high levels of stress related to both the transition to parenthood and the transition to a postpandemic society [2,24]. The findings of this study are consistent with these results overall. Specifically, the themes of *interpersonal troubles* and *financial help requests* that emerged are well aligned with previous studies, suggesting that stress was an affective state experienced widely by new and expecting fathers during the COVID-19 pandemic.

The results of this study also include a *miscellaneous* theme. Social media analyses are typically exploratory in nature; therefore, the researchers cannot predict what type of conversations will be uncovered. Although most captured posts will be directly related to the topic of interest, there is still potential to obtain *random* posts that are unrelated to the main topic. In this study, the miscellaneous theme included topics that were not directly related to new and expecting fathers. For example, Dur9 captured humorous responses; however, they were not necessarily related to fatherhood. This topic occurred only in the *during-pandemic* period, which could show an increase in humorous responses on the web. This use of humor in web spaces may be evidence of coping with the pandemic, as the world becomes more stressful to navigate and increasingly digital. It is well known that humor can function as a means of emotional regulation and act as a coping strategy for negative and stressful life situations [25-28]. More studies should be conducted to determine the effectiveness of humor as a potential health communication message appeal for efforts targeting expecting fathers.

### Implications

The findings from the study provide insight and guidance on strategies for both communication and public health professionals to better connect with new and expecting fathers on the web. Social listening analyses, such as the one conducted in this study, can be used to better understand the changing dynamics, behaviors, and needs of certain populations during rapidly changing events—such as pandemics and natural disasters—which can inform public health efforts in almost real time. For instance, the results of this analysis imply that fathers may use social media to cope with pandemic-related stress and seek advice related to fatherhood; therefore, public health officials should consider using the web to conduct campaigns and for interventions containing information related to parenting and stress management. In addition, these results suggest that online support groups and peer support interventions may be appealing to and safe for new and expecting fathers who are seeking enhanced social support as the world continues to battle the deadly COVID-19 pandemic.

Another useful finding for public health practitioners is understanding how external accounts are communicating with new and expecting fathers to promote baby-related information. Social media analyses are useful to see not only what a specific group is saying but also who is speaking to that group. For example, the *external parties targeting fathers* theme shows that social media accounts are targeting new and expecting fathers to promote a variety of baby products and articles with baby-related information. Further study of these posts and their levels of engagement can be useful for improving public health efforts that use social media to reach new and expecting fathers. Moreover, in almost real time, public health officials can use these types of social listening analyses to obtain a rapid assessment of the material and information needs that people who are pregnant, and their partners, may require during other health-related situations that call for immediate response such as abortion restriction, outbreaks of Zika virus, or natural disasters.

### Limitations and Future Studies

This study examined posts from both Reddit and Twitter; however, analyzing these 2 platforms together can present issues. These platforms provide different purposes and outlets for users, and the users and audience may have different characteristics. Although we searched for the same keywords in both platforms, users may divulge different information depending on the platform they are using. Relatedly, it is important to note that a portion of the social media posts that were captured cannot be definitively identified as coming from a new or expecting father.

There are also limitations to the data. First, the social media posts were limited to English-speaking users, resulting in the inability for generalizing the findings. Future studies should consider examining posts in other languages, such as Chinese and Spanish, as they are the second and third most commonly used languages, respectively, on the internet after English [29]. Future studies may also include alternative social networks, such as Weibo, to understand other samples. Second, the data set could not include every post related to new and expecting fathers, which ultimately limits the number of posts collected. Therefore, the results cannot reflect the entire population of interest. However, this study is exploratory in nature, and it is among the first to analyze Twitter posts for this specific audience. Future studies can apply this study’s insights toward quantitative research that is generalizable. The findings of this study can work toward future research that explores social media use among new and expecting fathers.

Another route of future studies includes a focus on emotions and sentiment analysis within social media analyses. Social media posts can correlate with the emotional well-being of an audience, and taking a deep dive into the sentiment of social media posts related to new and expecting fathers can provide a snapshot of this population’s well-being.

### Conclusions

With the onset of the COVID-19 pandemic, new and expectant parents faced unprecedented challenges amid a global pandemic. In addition, the current stream of literature that examines this group tends to give priority to the mothers, suggesting that fathers are overlooked or simply feel unprepared. Thus, this study sought to enrich research by focusing on new and expecting fathers, namely, by examining social media discourse related to this group both before and after the COVID-19 global pandemic was announced. Overall, the arrival of the COVID-19 pandemic appeared to have little impact on the excitement and resilience of new fathers as they transition to parenthood, in spite of considerable stress. These results speak to the immense resilience displayed by expecting fathers during the pandemic and imply that a major source of this resilience may stem from positive emotions that accompany becoming a parent. When communicating with new and expecting fathers, campaigns and interventions may consider appealing to the positive emotions and resilience that are commonly displayed during the transition to parenthood, as a means to promote prosocial coping behaviors among this unique population.

Altogether, these findings provide insight and guidance on the ways in which public health professionals can rapidly gather information about special populations—such as new and expecting fathers on the web—to monitor their beliefs, attitudes, emotional reactions, and unique lived experiences in context (ie, throughout a global pandemic). Insights gathered from studies such as these are enormously helpful for health communicators, as they think through the most efficient and effective ways to tailor messages for various audiences that, when taken together, comprise a specific population. Ultimately, we argue that public health officials engaged in health promotion should consider complementing traditional surveillance activities with social listening analyses, given the immense amount of heterogeneity that exists within communities and populations.

## Appendix

Multimedia Appendix 1 Boolean code used to collect social media posts.

Multimedia Appendix 2 Word cloud of topics from before the pandemic.

Multimedia Appendix 3 Word cloud of topics from during the pandemic.

Multimedia Appendix 4 Emotion in fatherhood posts according to volume.

## Abbreviations

RQ: research question
