# Supplementary material for: Web-Based Conversations Regarding Fathers Before and During the COVID-19 Pandemic: Qualitative Content Analysis
Source: JMIR Pediatr Parent. 2023 Feb 15;6:e40371. doi: 10.2196/40371 (PMC9978989; doi:10.2196/40371)
Supplement: Multimedia Appendix 1 [file pediatrics_v6i1e40371_app1.docx]

**Appendix 1.**

((#expectantdad OR #expectantfather OR #expectingdad OR #expectingfather OR #newdad OR #newfather OR #firsttimedad OR #dadtobe OR #fathertobe OR #becomingadad OR #becomingafather OR #imgonnabeadad OR #imgonnabeafather OR #imgoingtobeadad OR #imgoingtobeafather OR #newdadlife OR #expectingdadlife OR #newfatherlife)) OR (("i'm gonna" OR "I'm going" OR "I am" OR "I will" OR "I'm be") NEAR/10 ("be a dad" OR "be a father" OR "be the best dad" OR "be the best father" OR "new dad" OR "new father" OR "dad to be" OR "expect* dad" OR "expect* father" OR "first time dad" OR "father to be" OR "becoming a dad" OR "becoming a father" OR "new dad life" OR "becoming a papa" OR "becoming a pops" OR "becoming a daddy" OR "bun in the oven" OR "Turkey in the oven" OR "baby cooking")) NOT "I miss him greatly" NOT "Almighty" NOT "begotten" NOT "cops"
