# Supplementary figures and images for: Web-Based Conversations Regarding Fathers Before and During the COVID-19 Pandemic: Qualitative Content Analysis
Source: JMIR Pediatr Parent. 2023 Feb 15;6:e40371. doi: 10.2196/40371 (PMC9978989; doi:10.2196/40371)

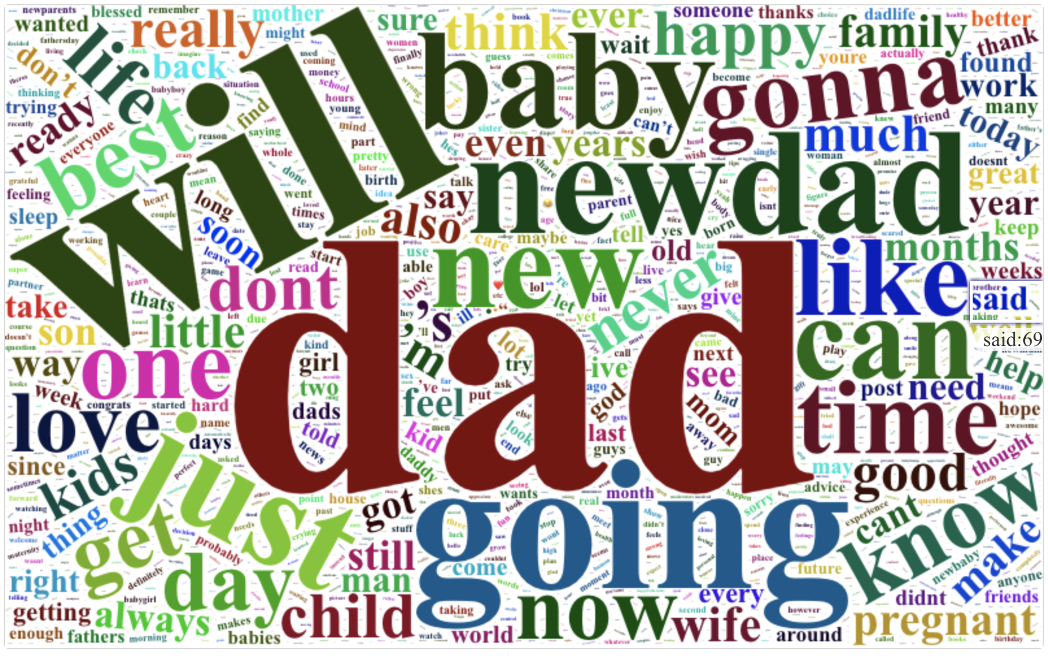

Supplement: Multimedia Appendix 2 [file pediatrics_v6i1e40371_app2.png]

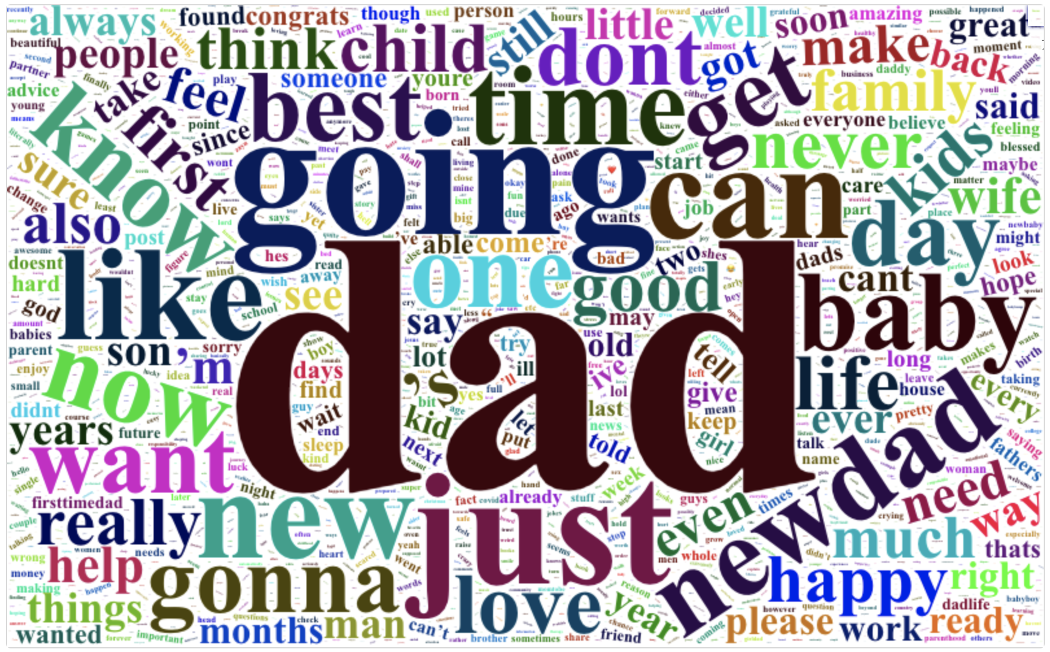

Supplement: Multimedia Appendix 3 [file pediatrics_v6i1e40371_app3.png]

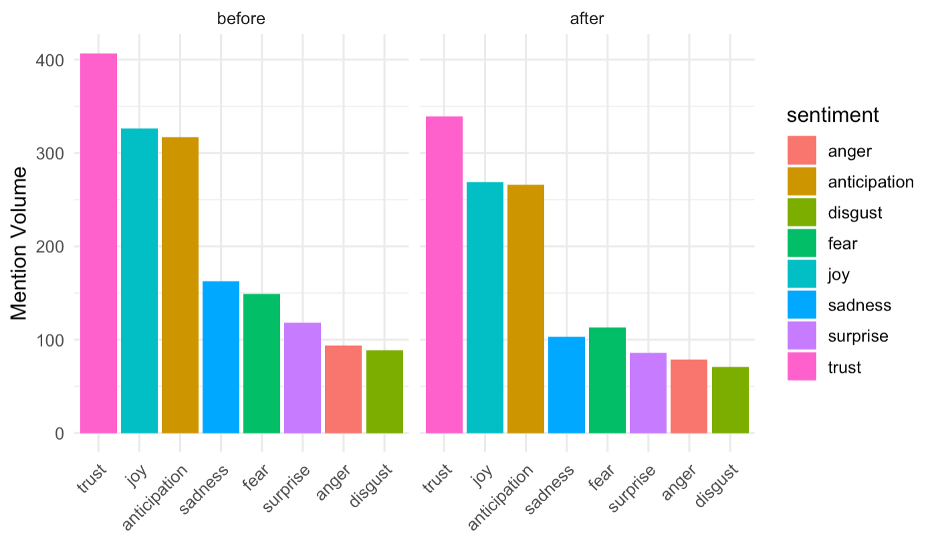

Supplement: Multimedia Appendix 4 [file pediatrics_v6i1e40371_app4.png]
